# Moyamoya Syndrome: Differential Diagnosis in Patients With Central Nervous System Symptoms and Hyperthyroidism

**DOI:** 10.7759/cureus.53519

**Published:** 2024-02-03

**Authors:** Yosuke Maezawa, Kazuya Nagasaki, Hitoshi Aiyama, Yuki Yamamoto, Yasushi Shibata

**Affiliations:** 1 Internal Medicine, Mito Kyodo General Hospital, University of Tsukuba, Mito, JPN; 2 Neurosurgery, Mito Kyodo General Hospital, University of Tsukuba, Mito, JPN; 3 Endocrinology and Metabolism, Mito Kyodo General Hospital, University of Tsukuba, Mito, JPN; 4 Neurosurgery, University of Tsukuba, Tsukuba, JPN

**Keywords:** hyperthyroidism, graves’ disease, carotid artery occlusion, moyamoya disease, moyamoya syndrome

## Abstract

Moyamoya syndrome, known as secondary moyamoya disease, is associated with various primary illnesses, such as brain tumor, meningitis, autoimmune disease, and thyrotoxicosis, and their relations are not clear. We report a rare case of moyamoya syndrome in a patient with Graves' disease. An 18-year-old woman was admitted to our hospital due to convulsions. She had symptoms of palpitations and fatiguability for half a year and transient numbness in her left upper extremity and dysarthria for a month. In physical findings, tachycardia and diffuse thyroid swelling were noted. A blood test revealed thyrotoxicosis and antithyroid antibody, and a diagnosis of Graves' disease was obtained. Brain magnetic resonance imaging (MRI) showed bilateral internal carotid artery occlusion. We finally diagnosed the patient with moyamoya syndrome caused by Graves' disease. Moyamoya disease or syndrome can cause symptoms like a stroke, sometimes requiring neurosurgical treatment. In our case, the therapy for Graves' disease resolved the symptoms. When diagnosing moyamoya disease, it is necessary to confirm whether there are any background diseases, such as Graves' disease.

## Introduction

Moyamoya disease (cerebrovascular "moyamoya" disease) causes chronic progressive narrowing in the terminal parts of both internal carotid arteries, forming an abnormal vascular network in the base of the brain, known as the "moyamoya vessels." Generally, it refers to cases without an underlying disease. However, cases with underlying diseases, including Graves' disease and systemic lupus erythematosus, are referred to as moyamoya syndrome [1,2]. The prevalence of moyamoya syndrome in patients with Graves' disease is reported to be 0.0454% [3], making it relatively rare. Therefore, we report a case of moyamoya syndrome associated with Graves' disease in an 18-year-old female.

## Case presentation

**Figure 1:**
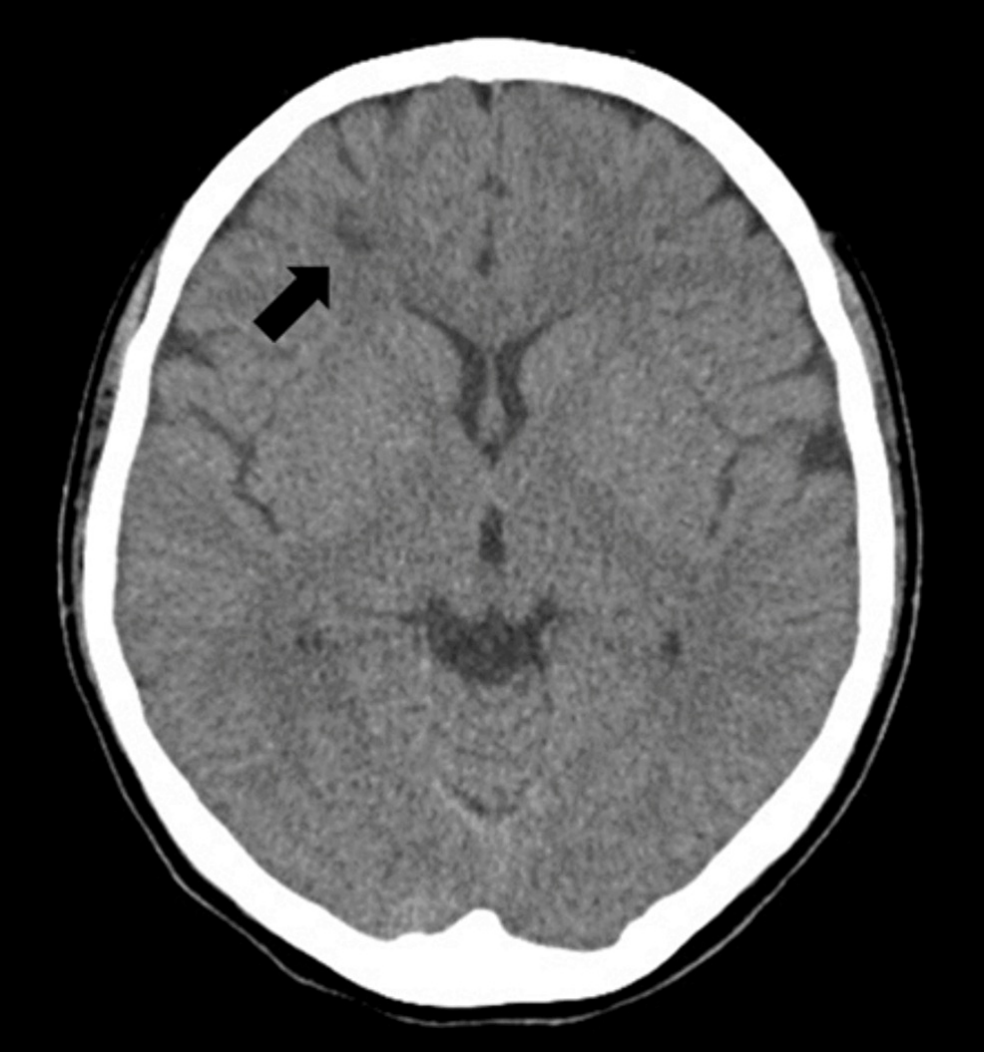
Computed tomography revealed two low-density areas in the right frontal lobe (black arrow).

An 18-year-old Japanese woman presented to our emergency department (ED) with a sudden loss of consciousness, which she regained after 15 min. She had experienced palpitations, increased appetite, and fatigue over the last six months. Physical examination revealed tachycardia (137 bpm) but no focal neurological symptoms. Cranial computed tomography (CT) revealed two low-density areas in the right frontal lobe (Figure 1). We suspected multiple sclerosis or brain tumors; therefore, the patient was discharged from the ED and scheduled for the outpatient clinic.

Subsequently, the patient developed generalized seizures and immediately returned to the ED. Diazepam (10 mg) and fosphenytoin (1125 mg) were administered intravenously, and the seizures stopped within five minutes. She had a blood pressure of 148/80 mmHg, a pulse rate of 118 bpm, a respiratory rate of 19 breaths per minute, oxygen saturation of 100% in room air, and a body temperature of 37.5°C. Physical examination revealed a goiter and blood tests showed elevated free T3 (>20.0 pg/mL) and free T4 (3.58 ng/dL) levels and low thyroid-stimulating hormone (TSH) levels (0.00 μIU/mL). The patient was admitted for the management of seizures and hyperthyroidism. A positive anti-TSH receptor antibody test confirmed Graves' disease and thiamazole was started. The patient experienced no convulsive or ischemic attacks and recovered to normal status during hospitalization. She was discharged on the sixth hospital day.

**Figure 2:**
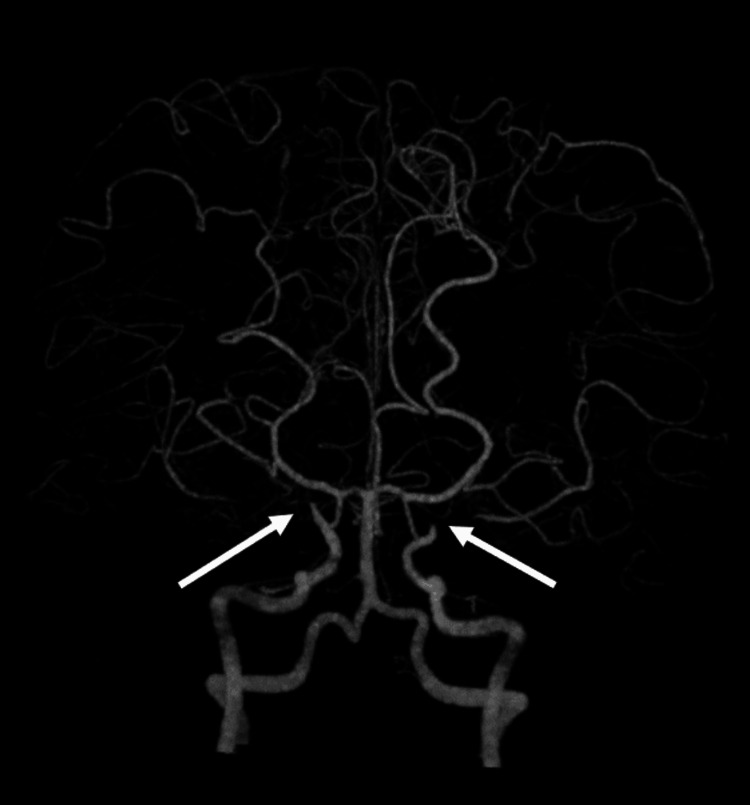
Magnetic resonance angiography showed obstruction of the bilateral internal carotid arteries (white arrows) and a hazy appearance in the surrounding blood vessels.

Post-discharge, cranial magnetic resonance imaging (MRI) revealed obstruction of the bilateral internal carotid arteries and a hazy appearance in the surrounding blood vessels (Figure 2), resulting in the diagnosis of moyamoya syndrome associated with hyperthyroidism. The two low-density areas in right frontal lobe detected in the brain CT at the ED were consistent with high signals on fluid attenuated inversion recovery (FLAIR), diffusion-weighted imaging (DWI), and T2-weighted images and low signals on T1-weighted images. From these findings, the lesions were suspected of being old infarctions. We suspected that her seizures and ischemic attacks were ischemic symptoms of moyamoya syndrome. We continued to treat Graves' disease and maintained her euthyroid status. There was no relapse of seizures in the year following discharge.

## Discussion

Moyamoya syndrome is associated with various congenital disorders and acquired diseases [1,2]. The prevalence of moyamoya syndrome in patients with Graves’ disease was reported to be 45.36 per 100,000 patients (0.0454%) [3].

Its clinical features can be divided into ischemic and hemorrhagic symptoms, clinically similar to primary moyamoya disease [4]. In moyamoya syndrome associated with hyperthyroidism, it may be challenging to differentiate moyamoya disease and thyroid crisis. These diseases present with central nervous system (CNS) symptoms, such as disturbance of consciousness and seizures. Despite that, in our case, no apparent end-organ damage was observed, indicating that the patient’s symptoms may have been associated with moyamoya syndrome and not thyroid crisis.

Previous studies have reported that hyperthyroidism may cause moyamoya disease because it increases cerebral oxygen consumption and blood flow, resulting in damage to blood vessel walls, sympathetic regulation causing cerebral vascular stenosis [5] and immune stimulation of the thyroid gland being involved in T cell function causing vascular abnormalities [6,7]. In moyamoya syndrome with Graves’ disease, it has been reported that normalization of thyroid function improves CNS symptoms and internal carotid artery stenosis [4,8,9]. On the other hand, there are also reports that the symptoms and artery stenosis, even if thyroid function becomes normalized and antiplatelet drugs or surgical revascularization is required [4,10-12]. In our case, there has been no recurrence of symptoms since thyroid function was normalized, and there was no need for antiplatelet drugs or revascularization of the internal carotid artery.

## Conclusions

Hyperthyroidism is one of etiologies of moyamoya syndrome; consequently, it is a potential differential diagnosis for Grave's disease, presenting with CNS symptoms. When a stroke occurs in a patient with Graves' disease, the coexistence of moyamoya syndrome should be suspected. Occasionally, this syndrome can be controlled with antithyroid medication alone.
